# “Worlds. . .[of] Contingent Possibilities”: Genderqueer and Trans Adolescents Reading Fan Fiction

**DOI:** 10.1177/15274764211016305

**Published:** 2021-05-19

**Authors:** Jennifer Duggan

**Affiliations:** 1 Norwegian University of Science and Technology, Trondheim, Norway

**Keywords:** fandom, gender nonconforming, identity, LGBTQIA2S, nonbinary, reader response

## Abstract

Cultural studies scholars have long been interested in the nexus between people’s online activities and their identities. One activity that has drawn attention is reading/writing fan fiction (fictions written by and for fans that build upon the characters and worlds depicted in commercial texts). While fan fiction and its surrounding communities have long been understood as resistant to heteronormativity, previous work exploring the fans who produce and consume fan fiction has largely insisted that most of these fans are adult ciswomen. Little has been written about the experiences of trans and genderqueer fans. To remedy this elision, this article explores two trans and genderqueer individuals’ experiences with fan fiction. It closely examines the roles reading, and especially reading fan fiction, has or has not played in their understandings of themselves, their identities, and their places in the world.

## Introduction

Fan cultures encourage participants “to think critically. . .and to make thoughtful and critical judgements about hegemonic culture” (Booth 2015, ¶ 1.1), including about how “identities are formed, subject positions made available, [and] social agency enacted” (Giroux 2004, 32). As popular culture may be “a significant pedagogical site that raises important questions about. . .subjectivity and experience” (Giroux and Simon 1988, 11), scholars of fandom have long been concerned with questions of “identity and representational politics” (Click and Scott 2018, 4).

A particular focus of fan scholars has been fan fiction—fan-authored texts which expand or reinvent worlds and characters from popular texts—and its surrounding digital communities. Because fan fiction transforms the works on which it is based and circumvents traditional gatekeepers, and because until recently, being a fan has been viewed, alongside other marginalized identities, as “deviant” (Jenson 1992), fandom often attracts marginalized populations and has therefore been envisioned as dissenting, resistant, and subversive (Jenkins 2018). Fan fiction thus creates opportunities for the ongoing lack of diverse commercially published texts (Tyner 2018) to be undermined and provides a critical pedagogical space in which marginalized fans can create their own “mirrors,” see through “windows,” or step through “sliding glass doors” (Bishop 1990) to engage with ways of identifying elided elsewhere.

Early work examining fan fiction focused almost exclusively on female fans (Click and Scott 2018; Jenkins 2018). While fan scholars have argued that the mainstreaming of fan cultures has rapidly diversified fan fiction communities (Click and Scott 2018; Hellekson and Busse 2006), binarized conceptions of gender still dominate discussions of fan cultures and behaviors. Although evidence suggests that genderqueer and trans populations are increasingly present in digital fandom (centreoftheselights 2014; Duggan 2020; McInroy and Craig 2018), they remain underrepresented in research.

This article explores the importance of fan fiction to two fans, genderqueer Beren Lemurian and trans John Holmes.^1^ Using participant-authored autobiographical narratives as data, the study emphasises the micropolitical, lived experiences of the participants to gesture toward gaps, ellipses, and elisions in our current understanding of who reads and writes fan fiction, and why. The article seeks to provide a platform for the genderqueer and trans voices thus far marginalized by fan studies. It aims to explore how these fans experience fan fiction and fan communities and to highlight the queer pedagogical potential of fan fiction and its surrounding communities.

The participants’ unique perspectives offer valuable insight into fan fiction’s affordances and the roles it may play in genderqueer and trans lives. Nevertheless, the study underscores the participants’ intensely personal, idiosyncratic engagements with fan fiction and aims to resist a reading of the participants’ experiences which reduces their engagement with fan fiction to using it as a plaster for the “problem” of queer identity formation in a heteronormative world.

## Fan Fiction, Genders, and Sexualities

That our identities are central to how we interpret and relate to texts is not a new concept (e.g., Bishop 1990; Busse 2017; Iser 1980; Rosenblatt 1978). While “those whose. . .identities fall within dominant groups find it easiest to forge identifications with. . .texts,” those who fall outside the intended audience may find relating to popular texts difficult, and thus may be more likely to read resistantly and transformatively (Jenkins 2018, 15; Tosenberger 2014, 7–8). Fan scholars have already highlighted the richness of reader response data available in fan communities, focusing in particular on fan fiction (Busse 2017; Jenkins 2013 [1992]), as fan fiction allows “people [to] share their fantasies, assert their identities, and negotiate change in their cultural environment” (Jenkins 2018, 23). Duncombe (2012) argues that the fictional worlds explored by fans allow them freedom to consider “norms, laws, and structures” and “imagine alternatives” (¶ 1). This is particularly important for “those considered a marginal audience,” including queer populations and “young people, who are. . .alienated from the means of production of the texts aimed at them” (Tosenberger 2014, 8). Queer young people are doubly alienated by commercially published texts (Tyner 2018), and it is thus particularly important for them to have the opportunity to engage transformatively with texts.

That speculative genres such as science fiction and fantasy have proven to be particularly popular objects of fandom will likely not come as a surprise: there is a rich history of scholarship demonstrating why the imaginative possibilities of speculative worlds invite subversion and resistance (Schalk 2018; Thomas 2019). Butler (2004) argues fantasy “is essential to an experience of one’s own body, or that of another, as gendered” (p. 15). For trans and genderqueer young people, speculative characters and narratives that challenge accepted discourses regarding bodies demonstrate how one can “knowingly negotiate the configuration[s] of [one’s] own body within a framework of subject formation” and thus fruitfully open up “shape-shifting possibilities” (Hurley 2014, 274–75). Butler (2004) asserts that fantasy “allows us to imagine ourselves and others otherwise” (pp. 28–29), while Sedgwick (1994) emphasises intense attachment “to a few cultural objects” as a mode of queer survival (p. 3). Fannish attachments can therefore deeply influence queer youths’ identities and sense of place in the world.

However, while fan scholars characterize identity politics as “the foundational and enduring influence” on fan studies (Click and Scott 2018, 4), a very specific nexus of identities has historically garnered the most attention. This nexus of identities is linked to “slash”—homoerotic and/or genderqueering fan fiction (Busse and Lothian 2014, 2018; Hellekson and Busse 2006; Lothian et al., 2007). Slash has arguably gained the most scholarly attention of any fan practice and is the focus of the works said to have founded fan studies (Bacon-Smith 1992; Jenkins 2013 [1992]; Penley 1991; Russ 2014 [1985]). These works characterize fan fiction communities as “overwhelmingly female” (Jenkins 2018, 19) and usually concern what has famously been called “normal female interest in men bonking” (Green et al. 2006 [1998], 61). They describe fans as “consciously producing their stories for the entertainment of other women, often. . .foreground[ing] their common experiences as women in a patriarchal society” (Jenkins 2018, 19). That these women were assumed to be mostly adult, straight, white, educated, middle-class, and able often went unremarked, in part because many aspects of diverse identities not only went unsaid in the “first wave” of fandom but were “unsayable,” either due to social constraints or because we did not have adequate terminology (Coppa 2014, 78). Nonetheless, the assumptions entangled within those foundational definitions have recently generated resistance from scholars and fans who consider themselves to be outside of this group.

One such consideration is age. The tendency in fan studies to view fan fiction-based communities as made up of “women in their thirties and older” (Lackner et al. 2006, 191) emphasises the “adult.” This dissociation of fannishness from youth relates to fan studies’ historical impetus to defend fans from accusations of juvenility (Jenson 1992; Tosenberger 2014). Nonetheless, examinations of fans in education and children’s literature studies often assume fans are young (Duggan 2017; Tosenberger 2008b, 2014), while recent work in fan studies has acknowledged the increasing presence of young people in digital fandom (Duggan 2020; Hellekson and Busse 2006; Jenkins 2006; Tosenberger 2014; Willis 2006). I have previously argued (Duggan 2020) that we ought to consider fan fiction communities as intergenerational spaces which blur the boundaries between “child” and “adult.” As Tosenberger (2014) suggests, many fandoms are “mixed-age,” with tremendous “potential for cross-generational interaction and. . .exchange” (p. 9; see also Jenkins 2006). Recent work has also drawn attention to the assumed whiteness of fans (Thomas 2019; Wanzo 2015) and the implied straightness of fans (Brennan 2014; Russo 2013, 2018). Such critiques demonstrate that we must “expand the range of familiar identity categories explored in the field” to “intervene in an already controversial, inevitably political endeavour involving emotion, identity, culture, and community” (Click and Scott 2018, 4, 3).

Fan studies’ foundational theoretical lens, feminism (Hannell 2020), has long been entangled with the queer (Love 2010) and included a focus on resistance to normativity, that is, on the queer as it is theoretically defined (Eng et al., 2005; Sedgwick 1994; Sommerville 2007). Some scholars have used queer theory as a framework for understanding the interactions between women in fan fiction communities (Lackner et al. 2006; Lothian et al. 2007), considered how writers use fan fiction to question hetero-/homonormativity (Duggan 2017; Lackner et al. 2006), or demonstrated how fan fiction makes visible queer reading practices (Brennan 2019; Duggan 2017, 2019; Tosenberger 2008a, 2008b). Particularly in the last decade, scholars have begun to explore the queer identities of fans and the characters they write (Brennan 2014, 2019; Busse and Lothian 2018; Driscoll 2006; Duggan 2017, 2019; Lothian et al. 2007; McClellan 2014; McInroy and Craig 2018; Russo 2013; 2018; Tosenberger 2008a, 2008b; Willis 2006, 2016), and scholars increasingly agree that “the fantasies of gender mobility and sexual freedom apparently played out in fan fiction may be really manifest” in fan fiction communities (Driscoll 2006, 86).

However, the growing number of studies examining queer individuals and communities in fandom focus mainly on the sexually queer (Brennan 2014, 2019; Lackner et al. 2006; Russo 2013, 2018), likely because queer theory sometimes privileges “homosexual ways of differing from heterosocial norms, and an antipathy (or at least an unthinking blindness) toward other modes of queer difference” (Stryker 2006, 7). Nonetheless, McInroy and Craig’s (2018, 184) survey of queer youths’ online activities and identities found that young people who participated in fandom were more likely to be queer, to have discovered this earlier in their lives, and to have nonbinary or trans gender presentations than queer youth who did not participate in fandom. Fandoms with high youth participation, such as the Harry Potter fandom (Jenkins 2006; Tosenberger 2014), appear to have a particularly high proportion of genderqueer and trans participants (Duggan 2020), and young fans are more likely to identify as queer than older fans (centreoftheselights 2014).

Even studies focusing on women acknowledge that “slash fandom veers much queerer than any representative cross-section of internet users” and that young fans, in particular, “describe. . .slash fan fiction as enabling them to explore and come to terms with their own queer identifications” (Busse and Lothian 2018, 124). That slash attracts a large trans and genderqueer readership is unsurprising considering its imaginative affordances (Busse and Lothian 2014; Driscoll 2006; Willis 2016). As Willis (2016) argues, slash emphasises that gender categories are “permeable; that sexual experience is translatable across bodies. . .; and that any individual—cis or trans—may. . .experience a gendered disjunction between their corporeal (literal) body and their felt (metaphorical) body” (p. 303). Indeed, Willis’s (2006) autoethnographic study of her experiences in the Harry Potter fandom demonstrates how slash can be reparative for the queer child, as it challenges “the queer-eradicating impulses” and “effacement of homosexuality” so common to commercially published texts for young people (p. 161).

But although it is clear trans and genderqueer individuals make up a significant proportion of fandom, and although it is also clear that the experience of being a fan can be particularly important mode of identity negotiation for young trans and genderqueer individuals, studies of their experiences are rare. The few studies which emphasise gender nonconformity tend to privilege readings of these bodies from the perspective of ciswomen, even as they admit the increasing diversity of fan fiction communities and argue for the political and personal importance of fan fiction to queer and trans readers (Busse and Lothian 2018, 124; see also Busse and Lothian 2014; Willis 2016). Welcome exceptions include McClellan’s (2014) use of trans theory to analyse material embodiment and gender identity in genderqueer and trans slash; Rose’s (2020) recent examination of “transfic as a digital form of (self-)narration” (p. 26); and Ledbetter’s (2020) theorization of “political dysphoria” (¶ 4.1–4.5). Nonetheless, there remains a scarcity of research focusing explicitly on genderqueer and trans fans’ interactions with fan fiction.

This paper centers young trans and genderqueer fans’ experiences. Because positive queer possibility models are often unavailable to young people (Rose 2020; Sedgwick 1994; Tyner 2018) and questions of identity linger longer, adolescence is here considered to extend into the participants’ twenties (cf. McInroy and Craig 2018; Reeson 2019). My aim is not simply to emphasise the experiences of a group whose voices have largely been elided within fan studies but also to respond to what I see as a problem in previous framings of fandom as a queer yet female space. Queer, by its very nature, resists binarized understandings of gendered behaviors and bodies (Eng et al. 2005; Sedgwick 1994; Sommerville 2007). I neither mean to argue that fandom *cannot* work as an exclusively feminist/feminine/female space for some nor to suggest that “female,” as a category, has not been envisioned in fan studies as a fluid and permeable term (Willis 2016); rather, I wish to emphasise that fandom can be multiple, often conflicting things simultaneously and that for those whose identities exceed or evade binary gender categories, it may hold meanings beyond those previously theorized. Fan fiction communities have for many become “a space to explore fabricated worlds that operate according to different norms, laws, and structures than those we experience in our ‘real’ lives” (Duncombe 2012, ¶ 1). Fandom is not only a subversive space in which binary gender categories are imagined as permeable but also a space in which identities *transcending* the binary are made visible and, thus, possible. If fandom is opposed to hegemony and the regimes of the normal, then it ought not also be solely subject to binarizing labelling. The elisions the “female space” label engenders invisibilize many fans.

## Methodology

This study uses written autobiographic narratives to gain insight into young trans and genderqueer fans’ entanglements with fan fiction. The use of participant-produced narratives as an exploratory-interpretive design for case studies is rare in fan studies; autoethnography is the privileged mode of in-depth qualitative inquiry (Hannell 2020). Autoethnography, however, has sometimes been critiqued because it emphasises behaviors privileged by academics (Hills 2002). Similarly, participant research that includes the academic’s voice, such as interviews, may nudge data in preferred directions. Participant-authored autobiographies, however, minimize unintentional researcher influence over the topics and details emphasised (Roth 2005).

Participants were selected using convenience sampling. Both were informed of the scope and intentions of the project, and both consented to participate. Their contributions have been anonymized. The first participant, John Holmes, “a twenty-nine-year-old PhD candidate in history,” is American, able, bisexual, white, and transmasculine. He describes his life as “divided between the US west coast and the UK” in urban environments. The second participant, Beren Lemuria, is an American, able, white, “thirty-four-year-old queer person, assigned male, with an unstable gender identity that floats somewhere in and around genderqueer.” They grew up in the “southeastern United States” and have lived throughout the country in urban and rural environments.

Each participant provided a written narrative of at least one page discussing (1) how they identified at the time of writing and (2) their engagements with fan fiction over time, including (a) when they read/wrote fan fiction, (b) what motivated them to read/write fan fiction, and (c) what aspects of fan fiction/fandom were most important to them. The narrative could take any form. They were given several months to write and were also given the opportunity to respond in writing to a late draft of this paper.

The participants’ written narratives (2,980 and 3,363 words in length, respectively) were analyzed to identify key emergent themes—*queer reader response, queer communities*, and *queer identities*—around which the results are organized. However, as the themes are heavily entangled in the data, clear-cut delineations between them are impossible.

The design of the study emphasizes the participants’ perspectives. The participants’ review of a late draft of the article reinforced perspectival faithfulness (Haraway 1988), recognizing Sedgwick’s (1994) assertion, “There are important senses in which ‘queer’ can signify *only when attached to the first person*” (p. 9, emphasis in original), and Butler’s (2004) argument, “Critique of gender norms must be situated within the context of lives as they are lived and must be guided by the question of what maximizes the possibilities for a liveable life” (p. 8). The focus of the study is strongly influenced by my own queer and fan affiliations.

## Results

### Queer Reader Response

Beren and John were both extensive readers in their youths. Beren describes their childhood home as a “bookish household”: “I’ve loved reading all of my life, helped run a bookstore for many years, and have been a writer for a long time.” They have always been drawn to fantasy fiction and, in their childhood, were particularly attracted to texts in which “a nerdy or socially outcast boy. . .finds entry into a secret world in which he discovers unforeseen capacities in himself and far more interesting people than he’d ever met.” John’s narrative similarly describes “phases of obsessive interest” with historical figures like Joan of Arc, “Scottish folk ballads,” and speculative texts like “Tamora Pierce novels,” *Star Wars*, and *Lord of the Rings* (LoTR). He emphasises that in adolescence, he never found texts describing people like him outside of fandom: “[fan fiction] presented me with possibility models at a time when those were virtually impossible to find offline.”

As such, Beren and John express differing relationships with books. For Beren, speculative fiction played a key role in providing imaginative horizons for their developing gender identity:

Fantasy and sci-fi have offered me more insight into my gender identity than queer theory and gender studies. . .It takes a radical break with the assumptions and constraints of reality. . .to think past the limits of gender and sexuality that make life so dreary. . .Reading [LeGuin’s *Left Hand of Darkness* and Clive Barker’s *Imajica*] helped me to reimagine androgyny, fluidity, cyclicality, and change over time in relation to my gender identity. . .[, as well as giving] me profound insight into how my genderqueer self gets read in social reality in the world I do live in.

John, however, was unable to find commercially produced texts that mirrored his gender identity; for him in particular, fan fiction appears to have provided important identity models. It is perhaps unsurprising, then, that John was not an active queer reader prior to encountering fan fiction given the early age at which he entered fandom; he was a preteen when he first encountered fan fiction and appears to have learned to read queerly through fandom.

John’s investment in fandom at this early age focused on LoTR. At twelve, he also published a fan fiction story of his own, which he describes as

an insane crossover about time travel and interdimensional hijinks involving characters from LotR, Star Wars, the Odyssey, medieval history, a Maoist cat, and a totally-not-self-insert character, a male Elder Being called Beatrice who was perpetually complaining about all the humans assuming he was a girl.

At fourteen, he “worked up the courage” to explore slash. He moved from reading Aragorn/Arwen to reading Aragorn/Boromir and Holmes/Watson. Then, in his later teens, John stopped reading slash in favour of “pretentious” texts, like Proust novels and Bergman films, but he returned to fandom in 2010 after an identity crisis. This return coincided with the release of BBC’s *Sherlock* (2010–17), and as such, he “found [him]self in the trans!Watson tag on AO3,” following the work of “two or three active writers.”

Beren’s fannish experience was quite different. While Beren “relish[ed] the homoerotic elements of [commercially published texts],” they describe themself as “never really [having] participated in ‘fan’ cultures relating to fictional series” until their early twenties. In their teens they were an “almost entirely solitary” fan and reader, but in their twenties, “a friend invited [them] to an all-night back-to-back viewing of all three extended LoTR movies,” after which they “rapidly got excited about LoTR,” read all of the Middle Earth-related texts and “a bio of Tolkein,” and listened “to a Tolkein podcast.” Around the same time, and although Beren had “grumpily refused to read” the Harry Potter novels out of a “disinclination to do what is socially trending,” they “grudgingly began to read them. . .and of course immediately loved them. . .[and] watched all the movies.”

Although Beren was unaware of fan fiction at this time, they particularly “relish[ed] the homoerotic elements of the LoTR films.” They point out that these popular fantasy texts were particularly appealing to them because “the dynamics [of the commercially produced texts] are already so shot through with homoeroticism.” Slash, once discovered, appealed because of its “straight up escapism.” Beren considers slash to be pure entertainment much of the time, but also acknowledges its value as a way “to explore [their] own fantasies and psychological dynamics through an entertaining lens.” They applaud slash’s “ability to materialize (in fantasy/writing, at least) homoerotic tendrils [they] sense beneath the surface of the world and the fictional representations [they] enjoy—queer wish fulfilment.” Beren read slash across a variety of fandoms and a variety of sites, starting with Harry Potter:

I enjoyed some HP/DM [Harry/Draco] stories, but went further afield into a wide range of pairings; Sirius/Lupin was a favorite (which I think is canon; also werewolfism in movie 3 is such an obvious homosexuality trope); Snape with Harry or other students or teachers; backstory pairings of Dumbledore with various others; and one of the hottest stories I ever read was Cedric Diggory with Viktor Krum, of all people . . . woof.

They later discovered other fandoms and pairings and diversified to reading Kirk/Spock, Holmes/Watson, and “one or two other fandoms,” but none “held [their] interest” quite like *Lord of the Rings*: “It’s LoTR slash that’s been my primary interest, and the Library of Moria where I’ve done the bulk of my slash reading.”

Beren also emphasises that reading slash reignited their passion for “fantasy genre fiction in [their] adult years”: “I’ve immersed myself in several other worlds with great gusto—most notably the Song of Ice and Fire world. . .But none led me to seek out fanfic; I was content with the exposition provided by the original author.”

It is important to note that neither John nor Beren read fan fiction as avidly now, in their thirties, as they did when they were younger. John states that he is “still close with a lot of people who are deeply embedded in fan communities. . .but my own involvement in consuming, and occasionally creating, fan content took place over two distinct phases in the past, each corresponding with a critical point in my slow, awkward process of Figuring Myself Out.”^2^ Beren, however, emphasises that while they “sometimes. . .read stories to masturbate, [they would]. . .more often. . .read them to *yearn*”:

For much of the time I was most immersed in reading slash, I either had only a long-distance lover(s) or no regular lover; fantasizing and yearning for romance. . .proved a source of great satisfaction. Slash characters provided a template for doing that, with particular aesthetic resonance and imaginative power.

### Queer Communities

John and Beren grew up in different environments, which shaped their adolescent experiences. John grew up in largely urban environments, giving him access to queer communities at a relatively young age, while Beren states that they grew up, “as most queers do, in a setting in which [they] knew hardly any other queer people and experienced many of [their] desires as weird, dangerous, tragic, or at least unlikely to be reciprocated.” Both were introduced to fan fiction through real-life friends: John, through “a friend at summer camp, who, over successive summers, was to become my first girlfriend and then my first boyfriend,” and Beren through a friend/lover, “a cis gay man. . .pretty monogamously focused on Harry/Draco [slash].” However, while Beren was very much a part of a knowingly queer community when this happened for them, John’s community was at first only liminally queer.

John used fan fiction communities as an exploratory space, and while he engaged with a fannish community related to his crossover fics, he did not connect with other fans of slash. He kept his two fannish identities fiercely separate: “There was the author of zany crossover fics, who commented and responded to comments, and then was the awkward porn-reading teen, who never commented or talked about the things [he] read.” Instead, he lurked, following “a number of gay men writing Holmes/Watson stories.” Their stories “gave [him] the guts to go out into the [local]. . .queer scene, first as a lesbian and then as a kind of awkward genderweird kid.” Similarly, in adulthood, fan fiction and fans’ blogs inspired John to connect with a real-life trans community in the UK, and he thus characterizes fan fiction as a “helpful first step” for his engagement with local queer communities. Nonetheless, he did not engage with other queer fans. While John does not characterize fandom as contingent on community, he asserts that community is important: “It’s not the same if you don’t have a group of people with whom you can correspond, share work, and share ideas.”

Beren, meanwhile, questions the idea of community as central to fandom for all fans. They took part in a number of real-life queer communities, both “activist” and “mainstream LGBTQ,” from their mid-teens (personal communication), and it was through these real-life queer communities that they were introduced to fandom, not the opposite. Despite their introduction to fandom through community, however, they emphasise that their fannish experience has been “almost entirely solitary”:

I didn’t seek out connection with other fans, attend any social events around fandom, etc. I consumed the books, movies, and other materials, and let my imagination run wild, mostly alone. I think this sets me apart from many fans for whom the experience of participating in fan culture is deeply social.

For Beren, this solitary exploration negated “the need to undertake *communal* identity exploration thru that medium [fan fiction] as many do” (personal communication, emphasis in original). Nonetheless, Beren argues,

I know that the standard received wisdom about who writes slash is that it’s heterosexual women who comprise the majority of authors and the bulk of readers, too. I have always been suspicious of this. . .My guess is that slash authorship and appreciation is far more queer and unpredictable than is commonly claimed. . .Most of the slash consumers I know are nerdy queer men and genderqueer folks.

### Queer Identities

Slash appears to have played a central role in developing John’s understanding of his gender identity and sexuality. John’s sexuality and gender identity shifted throughout his teens and early twenties. He highlights that he had little access to sources of knowledge about gender and sexuality when young. As such, fan fiction played an important role in his developing understanding of himself: “I may flippantly joke about how Sherlock Holmes made me bi and trans, but there’s a germ of truth there. . .I got my sex education (and miseducation) largely from the fan world.”

When John first started reading and writing fan fiction, he explains, “I was 12. . .I didn’t know that trans men existed or that there was a solution to the eldritch horror that was my mutating pubertal body.” Yet through fandom, he eventually came across and started to explore slash. This was “right around the time he decided (c. 14 or so) that [he] couldn’t really be bi.” Slash gave him “a more clear-eyed and realistic portrayal of what intimacy between two men looked like” than any other information source. Even when stories were unrealistic, “there was always somebody in the comments section who chimed in to point out that consent hadn’t been established well or, you know, that anal without lube was not going to be a good time for anybody.”

At first, thinking he was bisexual or a lesbian, John felt “a moral obligation to get into femmeslash,” but he found that “woman-woman scenes didn’t work for [him] in the same way” as gay male scenes. This led him to realize that the source of his internal conflict was not only sexuality but also gender. He reports that “boyfriend or not, [he] was pretty ambivalent about [his] attraction to men” throughout his teens (personal communication). It was in his early twenties that he admitted his “attraction to men was part and parcel of the realisation that [he]’d really only be able to live comfortably and enjoy intimate relationships as a man [him]self.”

John’s understanding of himself as trans thus took some time to develop, in part due to a number of complications in his questioning of his gender in his teens. When John first started to question his gender identification, his then partner transitioned from female to male. In the real-life queer community of which he was a part, too, “there were. . .trans men around but the ones [he] met presented as stereotypically masc straight guys.” John’s worry that he might be “just copying [his] partner,” combined with “a really uncomfortable conversation [with a trans man]. . .about testosterone and aggression[,] made [him] reluctant to espouse a trans male identity” in his teens. Nonetheless, he continued to present queerly.

When John moved to the UK for school in his early twenties, however, he “more or less went back into the closet.” This was largely because he felt unsafe, as “it’s far more common for middle-class liberals to openly espouse transphobic opinions” in the UK than it is in the urban centres of the American west coast. He began to present as “more femme” and label himself “a lesbian,” but after “a massive breakdown,” he “found [him]self turning to fandom again” for answers. Here, he found models of existence that seemed tenable:

The trans!Watson tag. . .was a mini-revelation. . .Their fics normalized the idea of being a trans man with a normal life. Not a genderqueer person, the identity I’d tried on as a high schooler. . ., but a trans man. . .I had better models for transmasculinity that wasn’t het and macho; for men who had intimate friendships with other men of the kind I’d had with my best guy friends but had always assumed would end if they ever saw me as a man. To this day I haven’t seen a single mainstream non-fan work that handles a character’s incidental transness as elegantly as some of those stories on AO3. . .These fics were also the first time I’d ever seen a description of a medically-transitioned male body in writing, presented as something both normal and desirable. . .Reading that story. . .made me realize really sharply that some of the parts I had could go away, and others could be understood and named differently by a partner. I could be re-embodied, and have someone else appreciate that body too.

These online communities also provided John with a window into the lives of real-life trans men—the authors of the stories he was reading. He was able to follow links to their personal Tumblrs. Two authors in particular stood out, one bi, the other gay: “Both were even more gender-non-conforming than I was, but they had still gone ahead and made the transition. It gave me hope.” While John never reached out to these authors and felt that “reading fan fiction could only take [him] so far,” the combination of slash and the Tumblrs of these men encouraged him to attend “an FTM Scotland meetup” and, ultimately, to transition.

Beren, too, has consciously identified as queer from their mid-teens, but throughout their childhood, they had “a sense of difference from others, and a suspicion (helpfully assisted by the gendered/homophobic bullying of others) that this might have had something to do with sexuality/gender” (personal communication). Their childhood was characterized by a sense of “longing for a secret world” and “kindred spirit[s]” (personal communication). As a child, then, they sought out speculative fictions because these fictions allowed them to find “modes of reimagining possibilities beyond the normative culture and categories immediately available,” as well as because speculative fictions often included “intense same-sex friendship/partnership” as a theme (personal communication). Beren was thus able to find imaginative possibility models by reading queerly even while they lacked a “vocabulary for making sense of [their] complex gender identity” (personal communication).

Nonetheless, the discovery of fan fiction in their twenties further expanded the imaginative and playful possibilities inherent in speculative fiction:

Perhaps one way to explain the significance of fan worlds and the erotic is the way that worlds inhabited by magic, mystery, epic purpose, unreal creatures and species, etc., seem radically contingent; they are not fixed by the familiar coordinate grid of possibility into which our IRL lives are squished. . .Inhabiting fan fiction worlds and thinking through the contingent possibilities they open up. . .help me rethink the gendered and sexual possibilities latent even in the (painfully unmagical, it sometimes seems) world I inhabit on a daily basis.

For Beren, fan fiction offers a space of imaginative possibility and speculation. While Beren is “engaged in an ongoing struggle to clarify [their] own gender identity, experimenting with different types of identity/presentation/embodiment, and also. . .lovers of many different genders,” they have nonetheless “identified almost exclusively with male characters” when reading fan fiction and have been “interested almost entirely in standard (homo) slash rather than het or femslash.” This has provided opportunity for reflection:

Most trans people I know. . .experimented with online cross-gender identification before coming out IRL, and many used fiction and identifying with cross-gender characters as a fantasy means of exploring the possibilities of identity. For whatever reason, this isn’t how I used slash or fan fiction. In a way, it may have been almost the opposite; through slash identifications, I was able to attain in fantasy a certain uncomplicatedly male and homo identification that’s never really corresponded to my lived experience over time.

## Discussion and Conclusion

These accounts bring into sharp relief a number of differences between how the participants developed their identities: Beren has identified as “different” since their childhood and continues to experiment with their gender and sexuality, while John feels quite secure in his presentation as a bisexual trans man and has a permanent partner (his fiancée). Beren was able to consider their identity through the imaginative possibilities of commercially published speculative fiction by reading what was “beneath the surface,” with slash “materiali[sing these]. . .homoerotic tendrils” and allowing Beren to “explore [their] own fantasies and psychological dynamics through an entertaining lens, and to inhabit different subject positions in doing so.” They insist speculative fiction and slash are valuable spaces in which “contingent possibilities. . .open up,” allowing them both to “rethink the gendered and sexual possibilities latent even in the (painfully unmagical)” real world and to find pleasure in “escapist fantasy” offering imaginative possibilities transcending cultural–material constraints. John, on the other hand, relied on slash to answer his questions regarding sexuality and gender: “I haven’t seen a single mainstream non-fan work that handles a character’s incidental transness as elegantly as some of those stories on AO3.”

Both John and Beren have used reading and fantasy to move “beyond what is merely actual and present into a realm of possibility, [to] the not yet actualized or the not actualisable” (Butler 2004, 28), in considering their genders, sexualities, and interpersonal relationships. This supports the assertions of critical work which suggests that speculative fiction is particularly attractive to marginalized readers and therefore encourages resistant and subversive readings (Hurley 2014; Thomas 2019), as well as work arguing that readers use texts as a basis for identification (Bishop 1990). Readers whose gender identifications fit uncomfortably within or transcend binarizing labels, who remain elided from texts for young people (Tyner 2018), must read “perversely” in order for texts “to remain powerful, refractory, and exemplary” (Sedgwick 1994, 4). As Beren’s account demonstrates, cross-identificatory fantasy can also have an important role, particularly when personal identity may be confusing or overwhelming. Cross-identification may allow individuals whose identities are fluid to experience a stability they do not feel in their daily lives. While cross-identification is also valuable to cisidentifying fans (cf. Willis 2016), imagined gender transcendence, cross-identification, and fluidity affect trans and nonbinary readers’ material, interior, and social lives.

Moreover, the above analysis demonstrates that real-life trans/queer communities and slash communities are deeply entangled. Slash can function as a gateway to real-life queer communities, and vice versa. For Beren, only the latter applied, but John’s experience appears to be a delicate interplay between both—slash and real-life function, for him, as a generative, mutually supportive feedback loop through which he was able to try on or experiment with various queer identities, including various iterations of transmasculinity and sexual attraction. Beren and John’s experiences demonstrate the very real confluences of off- and online trans/queer communities, despite that “the subcultural world of slash fan fiction writers can often seem highly removed from queer communities and practices” (Busse and Lothian 2018, 118). While this schism may be true for many straight, cisfemale fans, it is clearly less so for those whose lives are queer across their analogue and digital lives, including for ciswomen who participate in queer communities. The interconnectedness of digital and offline lives is central to both John and Beren. For John, digital fandom has functioned as an exploratory space and as a gateway to real-world sexual and gender expression, and in particular, provided transmasculine identities beyond those he encountered offline, while Beren has used slash to explore the dissonance between seemingly “uncomplicated” cis, homosexual desires and the more messy reality of their fluid identity and desires. Thus, while for many who maintain cis and/or straight lives outside of fandom, fan fiction offers a space for queer cross-identifications, it can also offer cross-identification flowing in the other direction, moving from the freedom and fluidity of a genderqueer life to what could perhaps be characterized as the security of cisgendered, homosexual relations.

While the narrative format of the autobiographies analysed here may seem to stabilize a reading of sexual and gender identity as linearly developed, I must emphasise that these accounts rather destabilize such an understanding. Both accounts demonstrate the transience, mobility, and vitality of our understandings of ourselves (cf. Sedgwick 1994), as well as revealing how popular cultural texts and the transformative readings of these shared by fans can act to both strengthen and undermine our identities. Moreover, while my focus here is on individual fans, I must stress that the outlook of these findings is a communal one. This is not limited to the offline trans/queer communities and kinship networks John and Beren discuss but also points toward a larger network of noncis fans: John underscores that while fandom gave him the courage to go in search of his local queer community, fandom in particular provided him with affinity models of transmasculine lives. Beren, on the other hand, argues that fandom provided them an understanding of themself—including how they might be seen by others—that they could not have developed offline. Both make clear that fan fiction is discussed in their local, offline queer communities; however, Beren’s and John’s engagements with digital fandom were solitary. For Beren, this appears to have been a conscious choice linked to the ways in which they prefer to consume texts, while for John, the choice to lurk was linked to his own uncertain gender identity. Nonetheless, both are “suspicious” of the “received wisdom” that ciswomen are the main group producing and consuming slash. Whether other trans, genderqueer, or nonbinary fans prefer to lurk rather than to interact with other fans bears further investigation. If this is a shared tendency, it may be one reason they have remained in the margins of fan studies, which tends to emphasize online community (Click and Scott 2018; Hellekson and Busse 2006).

This study is not without limitations. The small number of participants and the introspective nature of the data mean that the findings are not generalizable. Moreover, both participants are white, educated, middle-class, and able. This means that the article in some ways contributes to “transnormativity” (Stryker and Aizura 2013)—it makes visible “some trans [and otherwise genderqueer and nonbinary] bodies at the expense of others” (p. 4). In focusing on white, Anglophone, able, American individuals, this article elides several groups marginalized within fan studies, including transnational and multilingual fans, fans of color, and disabled fans.

The limitations of this case study, alongside its findings, act as points of departure for further research. As with transgender studies in general, within fan studies, a paper such as this one, with limited attention paid to intersectional concerns, may be “necessary to gain speaking positions within discourse” (Stryker and Aizura 2013, 3). I hope that further work can shed light on trans, genderqueer, intersex, and otherwise nonbinary identities in fandom at the intersections of (dis)ability, race, (trans)nationality, language, age, and class, as well as varied sexualities, echoing the impulses of scholars like Eli Clare, Kimberlé Crenshaw, Audre Lorde, José Esteban Munoz, and Eve Kosofsky Sedgwick. I further hope that this paper creates space for deeper discussions of gender and sexual fluidity, “mobility” (Driscoll 2006), and change, especially as they relate to fandom.

## Notes

1. Pseudonyms are used throughout.

2. John has returned to fan fiction since writing his autobiography (personal communication).
